# First mutation in the *FSHR* cytoplasmic tail identified in a non-pregnant woman with spontaneous ovarian hyperstimulation syndrome

**DOI:** 10.1186/s12881-017-0407-6

**Published:** 2017-04-26

**Authors:** Justine Hugon-Rodin, Charlotte Sonigo, Anne Gompel, Catherine Dodé, Michael Grynberg, Nadine Binart, Isabelle Beau

**Affiliations:** 1 Gynecology Endocrinology Unit, Port-Royal Cochin Hospital, University Paris Descartes, Paris, France; 2 Department of Reproductive Medicine and Fertility Preservation, Jean-Verdier Hospital, Bondy, France; 3 Inserm U1185, Univ Paris-Sud, University Paris Saclay, Le Kremlin Bicêtre, 94276 France; 4 Department of Biochemistry and Molecular Genetics, Cochin Hospital, Paris, France

**Keywords:** FSHR, Spontaneous ovarian hyperstimulation syndrome, Mutation, cAMP

## Abstract

**Background:**

Spontaneous ovarian hyperstimulation syndrome (sOHSS) is a rare event occurring mostly during natural pregnancy. Among described etiologies, some activating mutations of *FSH receptor (FSHR)* have been identified.

**Case presentation:**

We report hereby the case of a non-pregnant women with three episodes of sOHSS. Hormonal evaluation was normal and no pituitary adenoma was detected. However, genetic analysis identified a novel heterozygous *FSHR* mutation (c.1901 G > A). This R634H mutation is the first described in the cytoplasmic tail of the receptor. Functional analysis failed to reveal constitutive activity of the mutant but a decreased cAMP production in response to FSH. The weak activity of this mutant is correlated with a markedly reduced cell surface expression.

**Conclusion:**

Pathophysiology of non gestationnal sOHSS is still ill established. The molecular characterization of this new mutant indicates that it might not be at play. Therefore, further investigations are needed to improve knowledge of the molecular mechanism of this syndrome.

## Background

Ovarian hyperstimulation syndrome (OHSS) is an iatrogenic complication of supraphysiological ovulation induction in infertile patients. Evidence indicates that human chorionic gonadotrophin (hCG) plays a crucial role in the pathophysiology of this condition [1]. OHSS is characterized by ovarian enlargement due to multiple cysts together with abdominal distension. The pathogenesis of OHSS results in part from the production of several cytokines, probably enhanced by hCG, administered for final follicular maturation and further produced in case of pregnancy [2]. Clinical presentation is similar in spontaneous OHSS (sOHSS), a rare event mostly reported after natural pregnancy. sOHSS may result from high serum hCG levels encountered in multiple or molar pregnancies. However, Follicular Stimulating Hormone (FSH)-producing pituitary adenomas (FSHoma) or neuroendocrine tumors, activating mutations of the *FSH receptor* (FSHR), and hypothyroidism may also constitute risky situations [3, 4]. Five activating FSHR mutants have already been described in pregnant patients with sOHSS [5–9]. These gain-of-function mutations increase the sensitivity to hCG and/or to thyroid-stimulating hormone (TSH). By contrast, an impairment of FSHR function may cause severe folliculogenesis disorders such as ovarian failure [10, 11]. In the present paper, we report a case of non-gestational sOHSS associated with a novel *FSHR* mutation.

## Case presentation

**Fig. 1:**
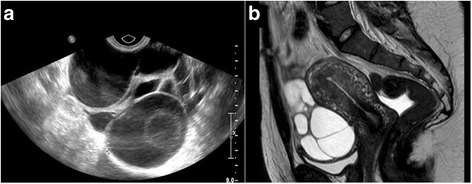
Multiple ovarian cysts revealed in pelvic ultrasonogaphy and MRI. Pelvic ultrasonography (**a**) revealed an enlarged 7x5 cm left ovary containing multiple cysts without fluid in the pelvis. This sagittal pelvic Magnetic Resonance Imaging (**b**) T2 weighted images showed a left multicystic ovary

A 26-year-old woman was referred for a medical treatment after 3 ovarian torsions. After spontaneous puberty, she had been naturally pregnant one time and after normal evolution, in particular without sOHSS, delivered one healthy baby. She used combined hormonal contraception (CHC) before and after pregnancy. Two years after the delivery, she was hospitalized 3 times in one year for pelvic pain. Laparoscopy was performed each time and showed torsions of adnexa with multicystic ovaries, while she used CHC. Finally, a right salpingo-oophorectomy revealed hemorrhagic infarction. One month later, pelvic ultrasonography revealed an enlarged 7x5 cm left ovary containing multiple cysts in the pelvis (Fig. 1a) confirmed by pelvic Magnetic Resonance Imaging (MRI) (Fig. 1b). After 6 months of treatment by gonadotropin-releasing hormone agonist, the size of left ovary was restored, without cysts. Etiologic assessment was performed during agonist treatment. Hormonal evaluation showed low serum estradiol (<43 pmol/L) and anti-Müllerian hormone (0.8 ng/mL) levels. Gonadotropin values were <0.5 UI/L for LH and 5 UI/L for FSH. Levels of prolactin, TSH, cortisol and androgens were in the normal range. No adenoma was detected on pituitary MRI. She reported that her mother who had two pregnancies never suffered from OHSS.

## Materials and methods

### DNA sequencing

Informed consent for DNA sequence analysis was obtained from the patient. DNA was extracted from peripheral blood leukocytes. All exons of the FSHR gene, together with intron-exon boundaries (around 10 pb) were sequenced using an automated sequencer (PGM life-technologies, AmpliSeq 3.4).

### Construction of mutated FSHR

The mutation was introduced into the pSG5-hFSHR plasmid [12] by oligonucleotide-mediated mutagenesis using QuickChange Site-Directed Mutagenesis Kit (Agilent). The construct was verified by Sanger method sequencing.

### Cell culture and transfection

COS-7 cells were grown with 10% fetal bovine serum in DMEM-F12 medium supplemented with L-glutamine and penicillin-streptomycin at 37 °C in humidified air containing 5% CO_2_. The cells were transfected with plasmids encoding the wild-type (WT) or mutated FSHR using the FuGENE 6 transfection reagent (Promega), according to the manufacturer’s protocol.

The efficiency of transfection was assessed by western blot analysis. Cells were lysed and total extracts were loaded onto 7.5% SDS-polyacrylamide gels. After transferring proteins on nitrocellulose membranes, blots were probed with FSHR323 monoclonal antibody [12] and anti-actin (clone C4, Millipore) antibodies.

### cAMP assay

Forty-eight hours after transfection, the intracellular accumulation of cyclic AMP (cAMP) was measured after incubation for 45 min with various concentrations of recombinant human FSH (Gonal-F®, Merck, France) using cAMP complete ELISA kit (Enzo Life Sciences).

### Immunofluorescence and confocal microscopy

FSHR323 was used to study, by indirect immunofluorescence, FSHR expression in transfected COS-7 cells as described previously [13]. For non permeabilized conditions, the antibody was applied on living cells for 1 h (h) at 4 °C in PBS containing 1% BSA, and the cells were fixed for 15 min (min) in 3% formaldehyde. After saturation with PBS/1% BSA the cells were incubated for 1 h with AlexaFluor 555 labeled anti–mouse antibody.

In some experiments, the cells were permeabilized for 4 min with 0.2% triton in PBS and incubated with FSHR antibody for 2 h at room temperature and further proceeded as non permeabilized cells. The samples were mounted in DAPI Fluoromont G medium (Southern Biotech). Imaging was carried out on a SP8 Leica confocal microscope.

### Surface immunoprecipitation of wild type and mutated FSH receptors

Cell surface expression of the wild type and mutated FSHR was analyzed by immunoprecipitation as previously described [14]. Briefly, FSHR antibody was added to the cell surface for one hour at 37 °C. Receptor-antibody complexes were extracted, purified and analyzed by western-blot.

### Statistical analysis

Data are expressed as means ± standard error of the means (SEM) and were analyzed with Prism software (GraphPad version 6.0) by using repeat measures ANOVA (Kruskal-Wallis test) to assess differences between groups followed by appropriated post-hoc comparisons (Dunn’s test). The p values lower than 0.05 were considered statistically significant. Experiments were performed a minimum of three times.

## Results

**Fig. 2:**
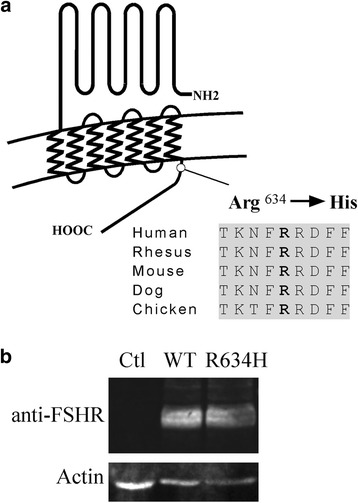
New FSHR mutation located in the beginning of the cytoplasmic tail and expression level of wild-type and mutated receptor after transfection. **a** Schematic representation of follicle-stimulating hormone receptor (FSHR) with the location of the R634H mutation in the beginning of the cytoplasmic tail and sequence alignment of amino-acid from chicken to human. **b** The expression level of wild-type (WT) and R634H in transfected COS-7 cells, is shown by western blot using specific antibody

Sequencing of *FSHR* gene revealed a heterozygous substitution of a guanine for an adenine in exon 10 (c.1901 G > A), resulting in the substitution of an arginine for histidine at position 634 of the protein (Fig. 2). The p.Arg634His variant (R634H) was referenced with allelic frequency of 0.000041 in the Exome Aggregation Consortium Browser Database. The Arginine 634, located at the beginning of the cytoplasmic tail, is highly conserved among species (Fig. 2a). *In silico* analysis revealed that p.Arg634His variant was predicted as “possibly damaging” and “deleterious” by Polyphen and SIFT respectively.

This variant was then introduced into an expression vector encoding the FSHR and was transiently transfected into COS-7 cells. The transfection efficiency of the WT and mutated FSHR, assessed by western blot, showed similar level of protein (Fig. 2b).

**Fig. 3:**
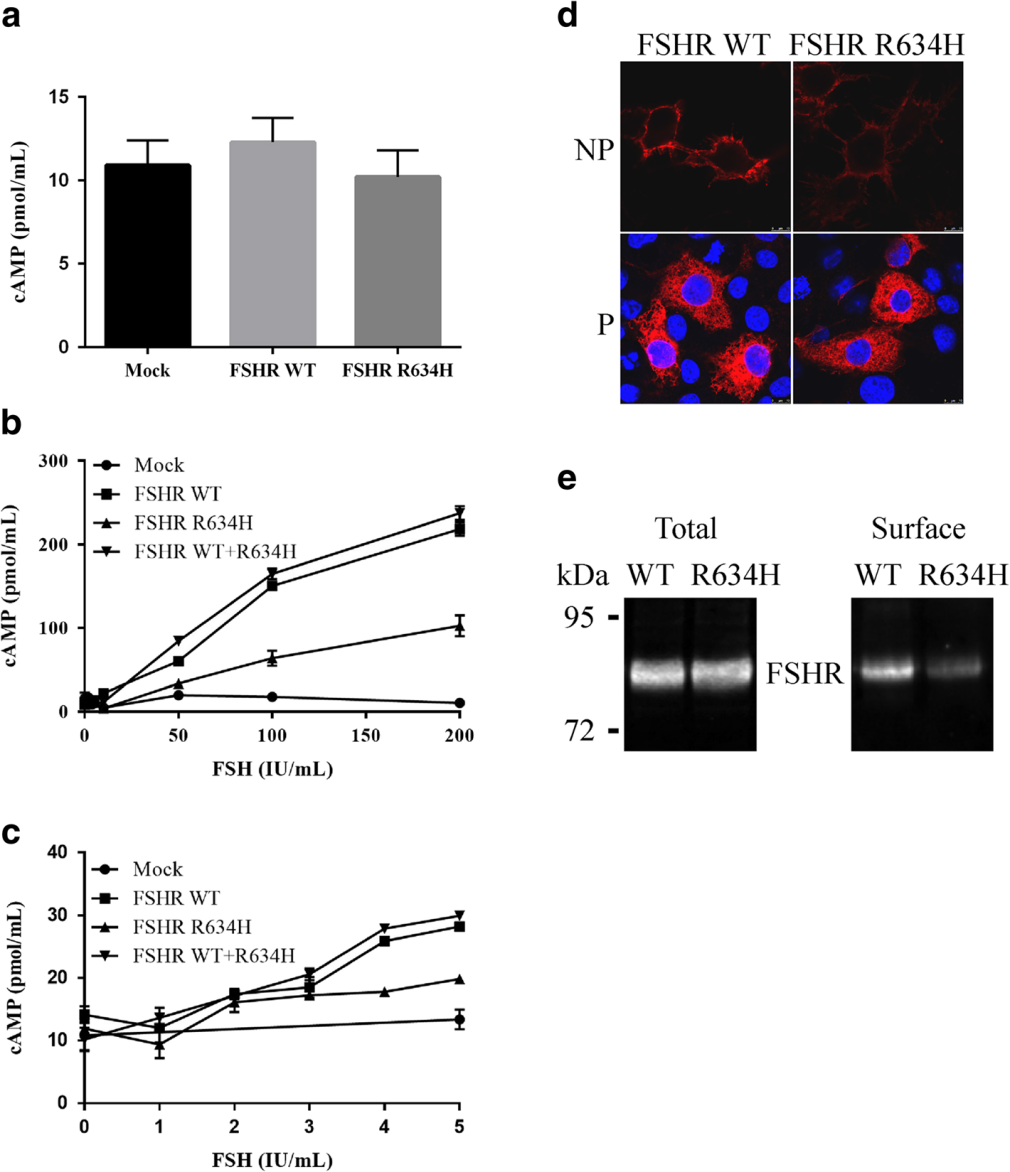
Functional studies of the wild-type and mutant FSHR. COS-7 cells were transfected with empty (mock) or expression vector encoding WT and/or mutated receptor. COS-7 cells were transfected with empty (mock) or expression vector encoding WT and/or mutated receptor. **a** Cyclic AMP (cAMP) level observed in transfected cells in the absence of any hormonal stimulation. Graph represents the results of at least three separate experiments performed in duplicate conditions. **b** and **c** Transfected cells were incubated with increasing concentrations of human follicle-stimulating hormone (FSH) and the accumulation of cAMP was measured. Each graph represents the results (mean ± SEM) of at least two separate experiments. **d** Cell surface expression of WT and mutated FSHRs. Permeabilized (P) or nonpermeabilized (NP) transfected cells were incubated with the FSHR323 antibody. Confocal microscopy was used to study the cellular distribution of receptors. **e** The membrane expression of the FSHR R634H is decreased. COS-7 cells were transfected with WT or mutated FSHR. Immunoprecipitation of FSHR from total extract (total) or from cell surface (surface) was performed using FSHR323. Immunoprecipitated FSHR was analyzed by western-blotting. Size of the molecular mass is indicated

To evaluate the functional activity of the mutant, we first analyzed the basal production of cAMP by the WT and mutated FSHR. COS-7 cells transfected with expression vector encoding receptor were incubated in DMEM containing 0.5 mM IBMX and the intracellular accumulation of cAMP was quantified. No significant difference in basal cAMP production by cells expressing the wild-type or mutated receptor was observed (Fig. 3a). COS-7 cells expressing either WT or R634H FSHR were then incubated with increasing concentrations of FSH. A cAMP dose-dependent response to FSH was observed in all conditions, but the response of the mutated receptor to high doses of FSH was decreased as compared to the WT. After transfection of COS-7 cells with equal quantities of the WT and mutant FSHR expression vector, mimicking the situation of heterozygous mutation, the response to recombinant FSH was similar to that of the WT receptor alone (Fig. 3b). In the absence of difference of response with high doses of FSH, response to physiological doses (1–5 mUI/mL) was analyzed. No difference between WT and mutated receptors was detected (Fig. 3c). In order to evaluate whether the mutated R634H mutant became sensitive to other ligands, stimulation with recombinant hCG and TSH were assessed. No accumulation of cAMP was observed in both conditions (data not shown).

To assess the cell surface expression of the mutant, the transfected cells permeabilized or not permeabilized were incubated with FSHR323 antiboby recognizing the ectodomain of FSHR. Confocal microscopy examination of permeabilized cells showed similar expression of WT and mutated receptors (Fig. 3d). Both WT and mutated receptors were observed at the cell surface in non-permeabilized cells, but the expression of the mutant was markedly reduced compared to the WT receptor (Fig. 3d). In order to quantify the WT and mutated FSHR at the cell membrane, we performed surface immunoprecipitation experiments. The level of R634H was decreased by 60% as compared to the WT FSHR while we detected the same amount of the two receptors in the total extract (Fig. 3e).

## Discussion and conclusion

Even though OHSS is mostly an iatrogenic complication, a consequence of hormonal stimulation in infertile patients [1], some cases may occur spontaneously, in particular during pregnancy [15]. Here, we report a case of sOHSS, resulting in three adnexal torsion, in a non-pregnant woman with normal gonadotropins and thyroid hormones levels and without pituitary adenoma. In our knowledge, just two cases of ovarian torsion had been described. The first one had been described during an episode of thyroiditis, in a patient with an history of two previous episodes of sOHSS during her two pregnancies [16]. The second one occurred in a patient with ectopic hypersecretion of FSH by a pancreatic tumor [4].

Today, only 5 heterozygous activating *FSHR* mutations have been described [5–9]. The common feature of these five mutations is that they have reduced specificity for FSH as they respond to increasing hCG levels that occur during normal pregnancy. Indeed, they become responsive to increasing hCG or TSH concentrations [5–9]. All of them are located either in transmembrane [5, 7–9] or in extracellular FSHR domain [6] and are associated with reduced specificity for FSH.

We described here the first mutation located in the FSHR cytoplasmic tail. The R634H residue belongs to the highly conserved BXXBB motif (where B represents a basic residue and X a non-basic residue) located in the receptor cytoplasmic tail [17]. When the mutant *FSHR* was transfected into COS-7 cells in the absence of ligand, no constitutive activation was observed. Moreover, there was a decrease in cAMP production after FSH administration compared to WT and surface cell expression was impaired. These results are in accordance with several studies evaluating the functional role of the cytoplasmic tail of FSHR and particularly the BXXBB motif [17–19]. Indeed, functional analysis revealed that the cytoplasmic-tail of FSHR plays an indispensable role in cell surface receptor trafficking [20] and BXXBB is particularly important for membrane expression [17, 18]. Moreover, we showed that in heterozygous condition, the response to recombinant FSH was similar to that of the WT receptor alone. In the same way, Zariñán et al. had reported that co-transfection of wild type FSHR with mutant FSHR in the BXXBB of the cytoplasmic tail showed dose-dependent inhibition in cAMP production with increasing amounts of mutant DNA and subsequently rescue of function by co-transfection with wild-type fragments suggesting oligomerization of FSHR [21].

We report, in a young patient suffering from recurrent and unexplained sOHSS, the first *FSHR* mutation located in the cytoplasmic tail of the receptor. However, contrasting with the phenotype, functional analysis demonstrated that this mutant does not exhibit any constitutive activity. Similarly another reported FSHR variant, M512I, also found in non-pregnant woman with sOHSS, did not demonstrated her involvement in this pathology [22]. Nevertheless, both case reports highlight the importance of performing functional analysis to ensure that new *FSHR* mutations are actually at play in sOHSS. Further investigations and genetic analysis will be needed to understand the pathophysiology of sOHSS.

## Abbreviations

cAMP: cyclic Adenosine MonoPhosphate
CHC: Combined hormonal contraception
DNA: Deoxyribo nucleic acid
FSH: Follicular stimulating hormone
FSHoma: FSH-producing pituitary adenomas
FSHR: FSH receptor
hCG: Human chorionic gonadotrophin
LH: Luteinizing hormone
MRI: Magnetic resonance imaging
OHSS: Ovarian hyperstimulation syndrome
SEM: Standard error of the means
sOHSS: Spontaneous OHSS
TSH: Thyroid-Stimulating hormone
WT: Wild-Type
